# Supplementary material for: Cellular and subcellular heterogeneity of astrocytic Na⁺ homeostasis tuning astrocytes into functionally distinct subgroups in the mouse brain
Source: Nat Commun. 2026 May 20;17:4515. doi: 10.1038/s41467-026-73435-z (PMC13190688; doi:10.1038/s41467-026-73435-z)
Supplement: Supplementary file 1 — Supplementary Information [file 41467_2026_73435_MOESM1_ESM.pdf]

Supplementary Information

Cellular and subcellular heterogeneity of astrocytic Na^+ homeostasis tuning astrocytes into functionally distinct subgroups in the mouse brain,

Meyer et al.

<u>Supplementary Figure 1:</u>

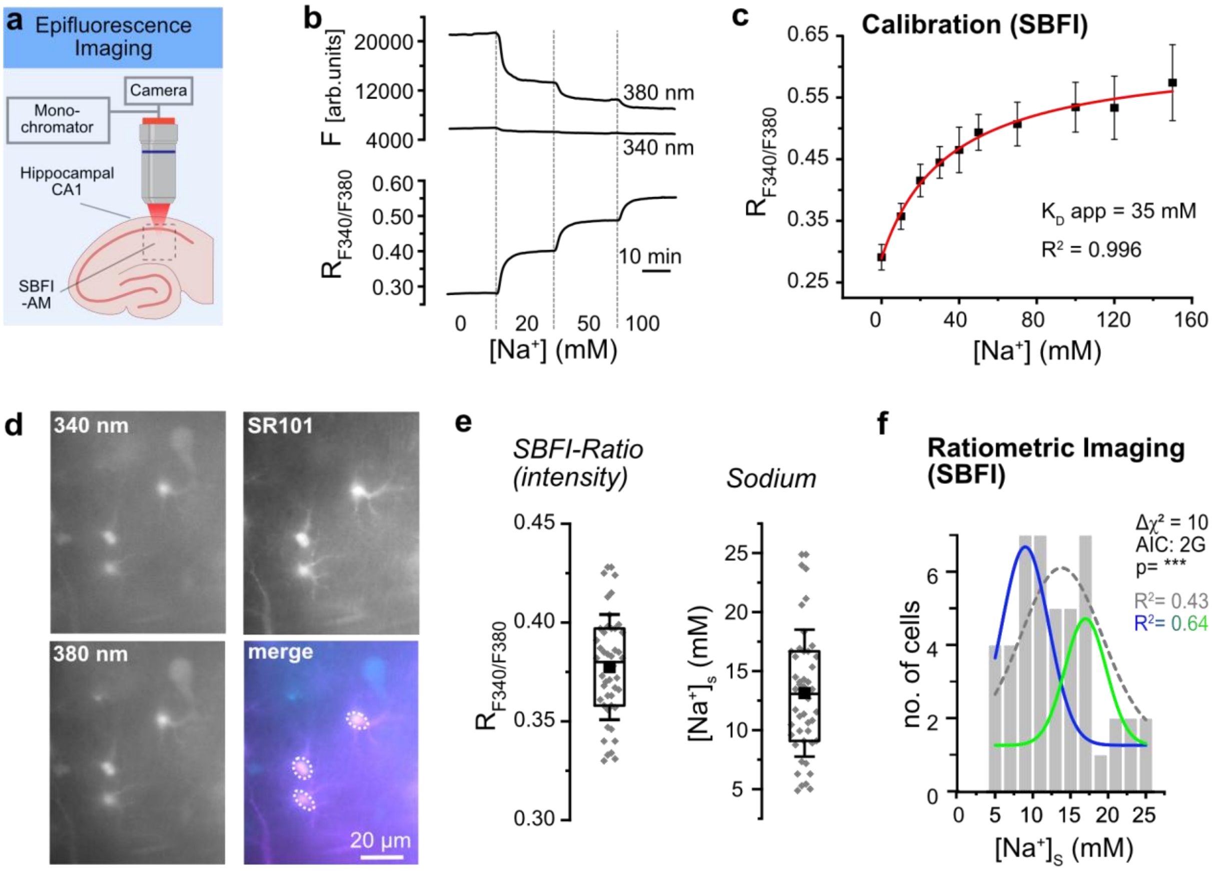

Suppl. Fig. 1: Intensity-based determination of astrocyte $[Na^+]_S$ in the hippocampal CA1 area. a Scheme of experimental design. **b-c** *In situ* calibration of SBFI fluorescence in cellular somata. After SR101-labeling and subsequent bolus-staining with SBFI-AM, slices were perfused with calibration salines containing ionophores and 0-150 mM Na^+ (n=508, N=9 for each $[Na^+]$). **b** Changes in somatic SBFI fluorescence at 340 and 380 nm excitation (top) and the calculated fluorescence ratio ($R_{F340/F380}$; bottom) upon changing $[Na^+]$ as indicated (n=84 neurons and astrocytes, traces smoothened using a rolling average filter (10 points)). arb. units: arbitrary units. **c** Relationship between fluorescence ratio ($R_{F340/F380}$) and $[Na^+]$. The red line represents a Michaelis-Menten fit, revealing an apparent K_D of 35 mM. **d** Epifluorescence images of the CA1 *stratum radiatum* after staining with SBFI-AM (340 nm/380 nm excitation) and SR101, as well as merged image of SBFI (380 nm, cyan) and SR101 (magenta). Dotted lines indicate somatic ROIs from which fluorescence was analysed. **e** Bar charts illustrating SBFI fluorescence ratio ($R_{F340/F380}$) (left) and corresponding $[Na^+]_S$ (right) in astrocytes in standard ACSF. **f** Distribution of astrocyte $[Na^+]_S$. Blue and green lines represent a double-Gaussian function, grey dotted line is a single-Gaussian function. $\Delta\chi^2$ (ΔChi^2), AIC (Akaike information criterion), 2G (2 Gaussian), R^2 (coefficient of determination) are as indicated. **c, e** Diamonds: individual data points, boxes: 25/75, whiskers: SD, black lines: median, squares: mean. **e, f**: n=46, N=8. Further details on statistics are provided in the results and statistical summary file. Source data are provided as a Source Data file.

Supplementary Figure 2

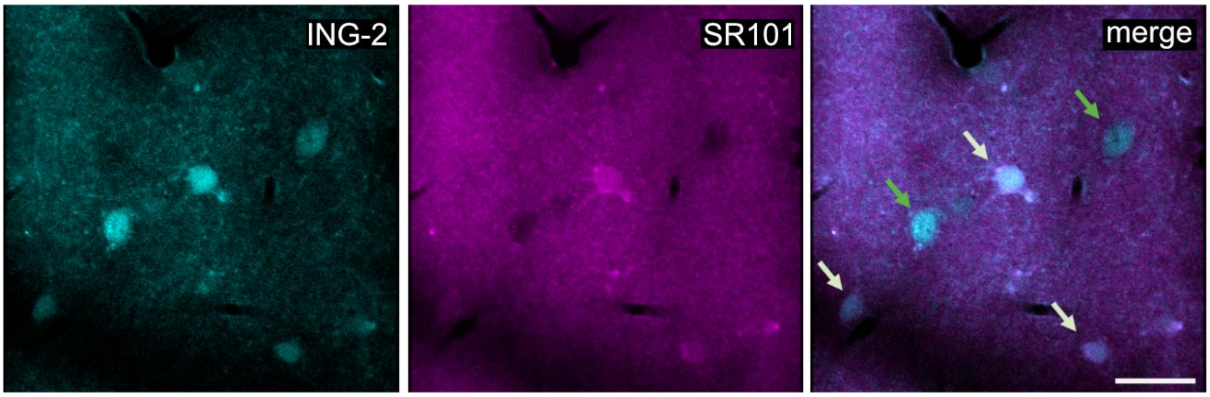

Suppl. Fig. 2: Identification of cortical astrocytes and neurons *in vivo*. Representative two-photon excitation fluorescence images of layer 2/3 cortical cells labelled by ING-2 (left panel, cyan) and SR101 (middle panel, magenta) *in vivo*. Right panel: merged view, white arrows indicate SR101-positive cells (i.e., astrocytes) whereas green arrows indicate SR101-negative cells (i.e., neurons). The images were acquired in resonant-galvo scanning mode (10 Hz frame rate), each image is the average of a 40-frame intensity stack. Scale bar: 25 µm.

Supplementary Figure 3

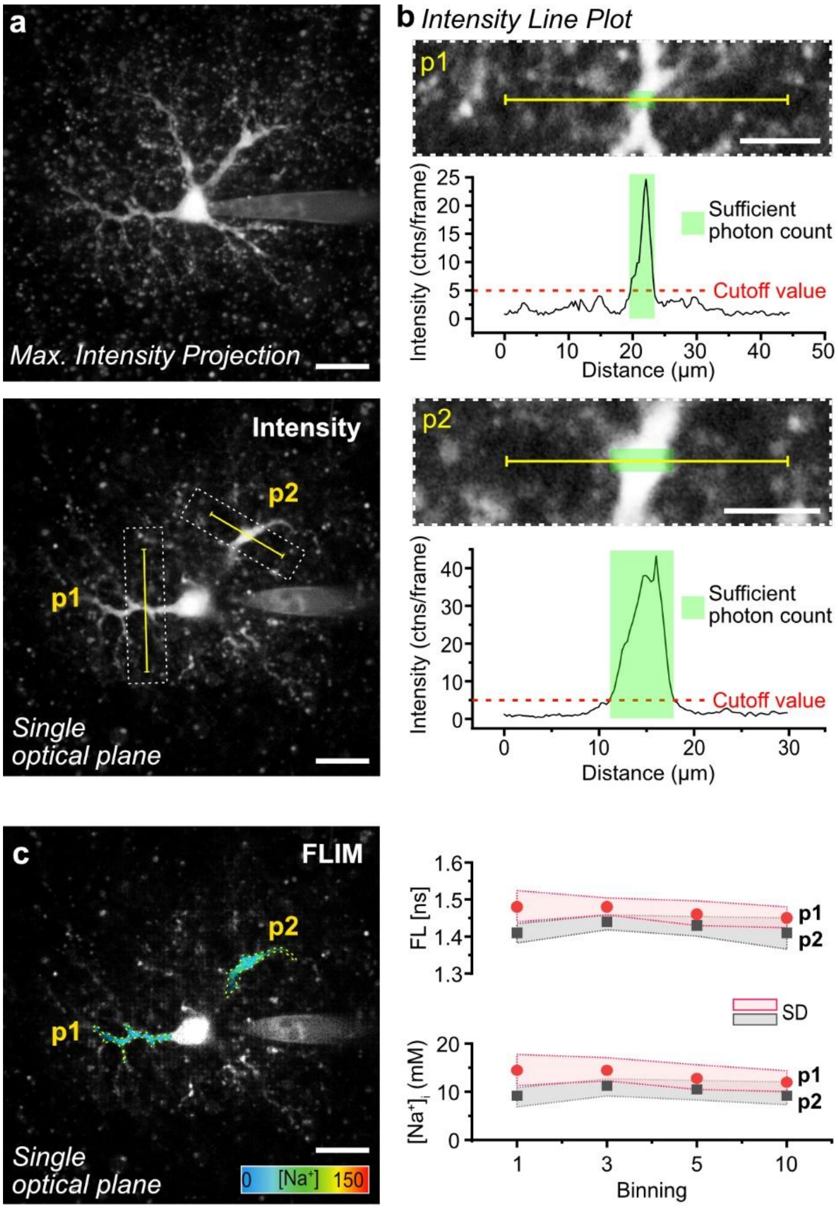

Suppl. Fig. 3: Minimum photon count for ROI selection and influence of binning on FL calculation: a Maximum intensity projection of a z-stack of an astrocyte loaded with ING-2 via whole-cell patch clamp (top) and image of a single optical plane of this z-stack (bottom). Dotted boxes outline two processes (p1, p2), shown in b at higher magnification, yellow lines indicate the lines from which the intensity plots were taken. **b** Images of p1 and p2 at higher magnification. Note that their brightness was increased twofold for better visualization of the gliopil surrounding the main processes. The yellow lines depict the lines from which the photon counts were determined as shown in the traces below. Green overlays indicate structures, in which the photon count was ≥5 photons per frame per pixel, red dotted lines mark the cutoff value. **c** FL image of the same optical plane as in a (bottom). p1 and p2 are color-coded ROIs from which FL was determined as shown on the right. Right: FL (top) and calculated $[Na^+]_P$ (bottom) of the two processes at different temporal frame binning (1-10 frames). Overlays indicate the standard deviations of the respective FL fits. Note that binning did not alter FL and $[Na^+]_P$. Further details on statistics are provided in the results and statistical summary file. Source data are provided as a Source Data file. a, c Scales: 20 µm. b Scales: 10 µm.

Supplementary Figure 4

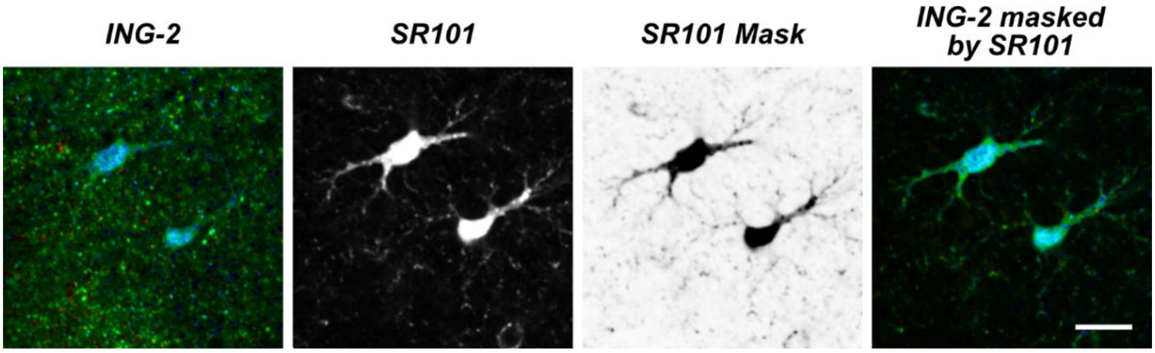

Suppl. Fig. 4: Masking ING-2 fluorescence lifetime with SR101 intensity to improve visualization of processes. From left to right: ING-2 fluorescence lifetime (FL) image, SR101 intensity image, inverted SR101 intensity image employed as a mask and ING-2 FL image masked by SR101. Utilizing the SR101 intensity image to mask the ING-2 LT image results in greatly increased visual perception of astrocyte processes. Scale: 10 µm.

Supplementary Figure 5

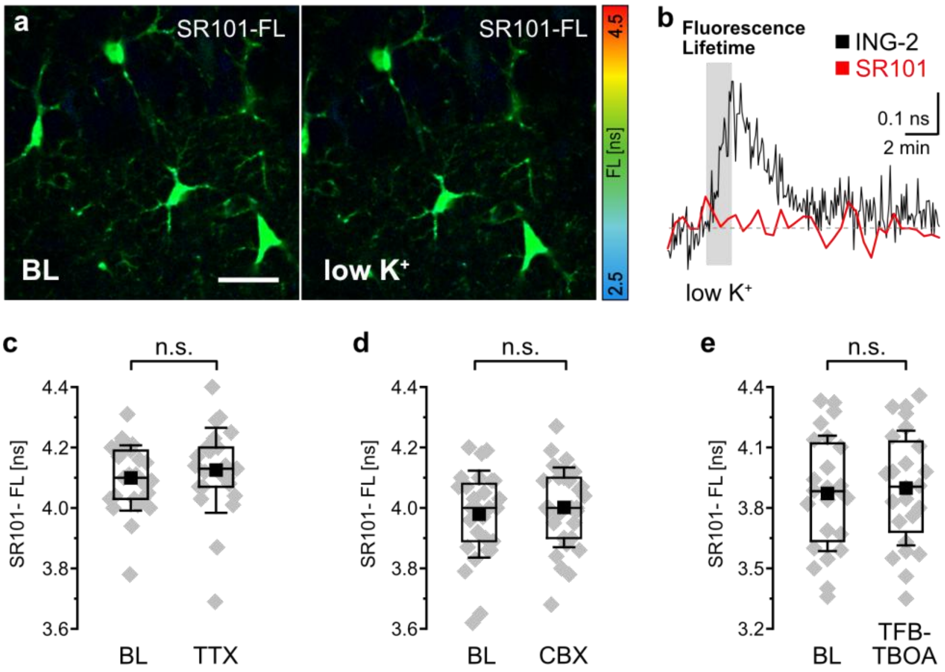

Suppl. Fig. 5: Influence of different drugs on FL of SR101. a Color-coded images of SR101-FL of astrocytes in baseline conditions (left, BL) and at the peak $[Na^+]_S$ increase induced by a 2-minute perfusion with low K^+ (right). Note that the images are virtually identical. Color-code is depicted on the right. SR101-FL was calculated using rapid reconvolution (see Methods) and an unfixed monoexponential decay. Scale bar: 20 µm. **b** Exemplary traces of an individual astrocyte depicting changes in FL of ING-2 (black) and SR101 (red) induced by low K^+ (grey shaded area). Notably, while ING-2 FL changes by ~0.4 ns (corresponding to an increase in $[Na^+]_S$ by ~80 mM), no change in SR101-FL can be observed. **c-e** SR101-FL in baseline conditions (BL) and during perfusion with 0.5 µM TTX (BL: n=27, TTX: n=25, both N=5; p=0.46) (**c**); 100 µM CBX (BL: 29, CBX: n=30, both: N=4; p=0.54) (**d**); and 1 µM TFB-TBOA (both: n=24, N=5; p=0.22) (**e**). Diamonds: individual data points, boxes: 25/75, whiskers: SD, lines: median, squares: mean. Further details on statistics are provided in the results and statistical summary file. Source data are provided as a Source Data file.

Supplementary Figure 6

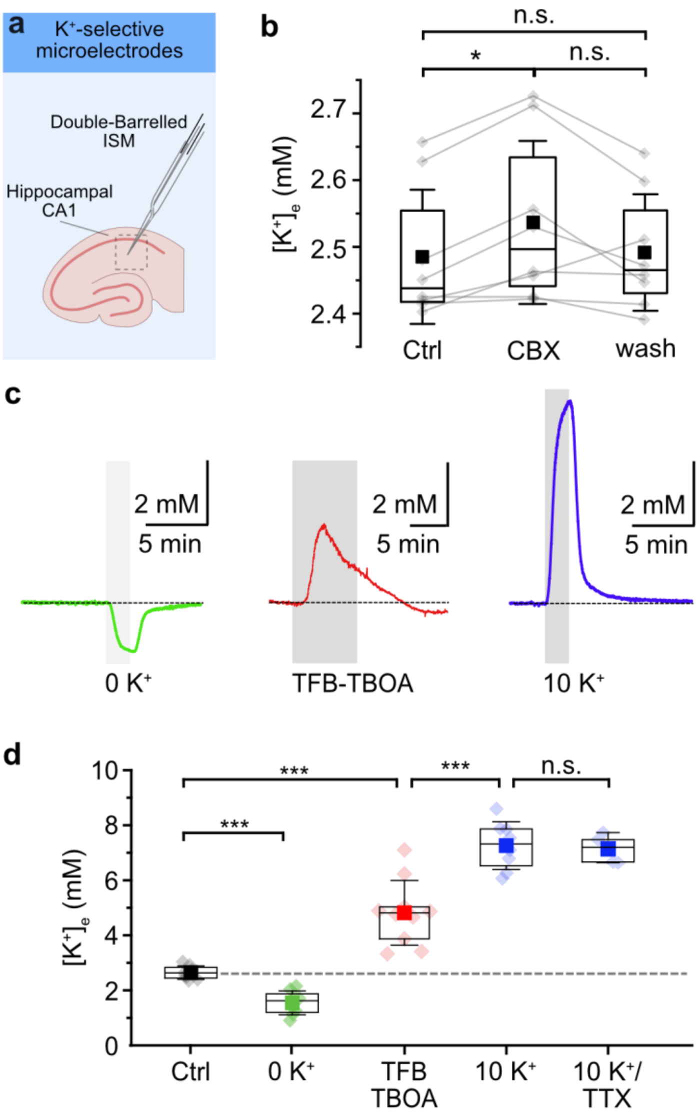

Suppl. Fig. 6: Changes in $[K^+]_e$ in the CA1 *stratum radiatum* determined using double-barrelled ion-selective microelectrodes. a Scheme of experimental design. **b** $[K^+]_e$ at baseline (Ctrl), during perfusion with CBX, and after recovery (wash) (N=8). Lines connect data points from individual measurements/slices. Note that $[K^+]_e$ increases significantly during CBX (p=0.016), and partially recovers after wash-out of the drug (p=0.064). **c** Exemplary traces showing changes in $[K^+]_e$ induced by perfusion with K^+-free ACSF (0 K^+), 1 µM TFB-TBOA and ACSF containing 10 mM K^+ (10 K^+) as indicated by grey shaded areas. **d** Bar charts illustrating $[K^+]_e$ in standard ACSF (Ctrl; n/N=8) and peak values induced by 0 K^+ (N=8; p=2.74E-06), TFB-TBOA (N=10; p=1.83E-04), 10 K^+ (N=8; p=1.70E-04) and 10 mM K^+ in the presence of TTX (N=5; p=0.77). **b, d** Diamonds: individual data points, boxes: 25/75, whiskers: SD, lines: median, squares: mean. Further details on statistics are provided in the results and statistical summary file. Source data are provided as a Source Data file.

Supplementary Figure 7

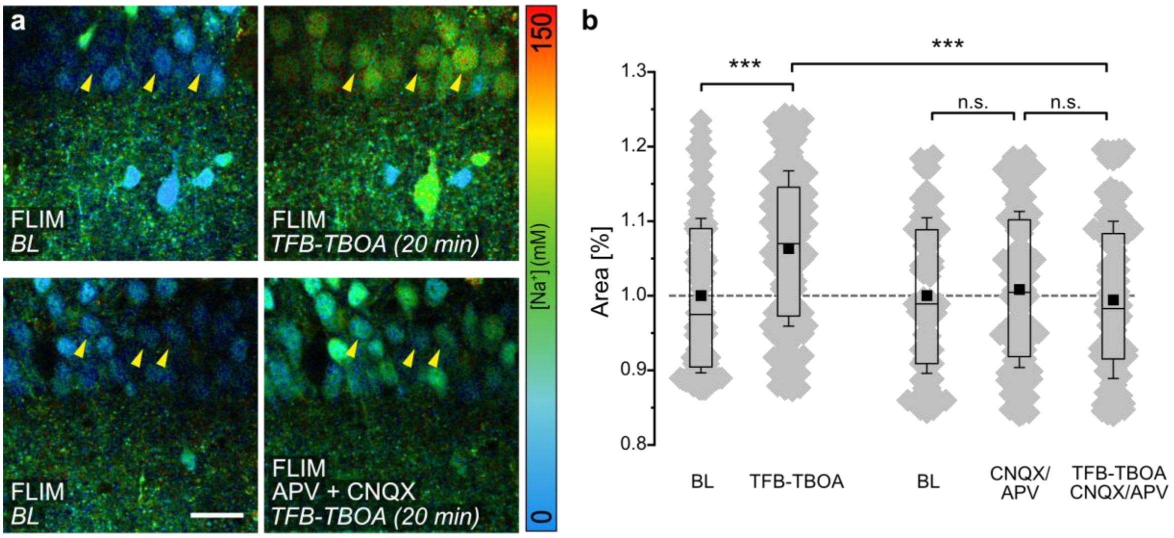

Suppl. Fig. 7: Relevance of ionotropic glutamate receptors in TFB-TBOA-induced neuronal swelling. a Top: Color-coded FL images of ING-2-stained CA1 pyramidal neurons and astrocytes in the *stratum radiatum* in baseline conditions (BL) and 20 minutes after perfusion with 1 µM TFB-TBOA. Bottom: Same experiment with CNQX (10 µM) and APV (50 µM) added ≥15 min prior to TFB-TBOA to block AMPA and NMDA receptors, respectively. Arrowheads highlight swelling and rounding up of neuronal somata in TFB-TBOA (top), which was blocked in the presence of CNQX and APV (bottom). Note that at the same time, the TFB-TBOA-induced increase in neuronal $[Na^+]_S$ was dampened by the receptor blockers. Color-code for $[Na^+]$ is depicted on the right. Scale: 20 µm. **b** Automated area analysis of ING-2 fluorescence intensity of neuronal somata using the software DL-SCAN (1) for automatic, deep-learning-based selection of ROIs. ROIs with an area of <50 μm^2 and >100 μm^2 were excluded from analysis. The box plots show that the somatic area increased significantly by 6±10% after 20 min of TFB-TBOA (BL: n=117, N=6; TFB-TBOA: n=138, N=6; p=2.54E-06). With receptor blockers present, no swelling was observed (BL: n=76, N=4; CNQX/APV: n=104, N=4; p=0.60 / TFB-TBOA/CNQX/APV: n=101, N=4; p=0.71; vs. TFB-TBOA: p=2.41E-06). Diamonds: individual data points, boxes: 25/75, whiskers: SD, lines: median, squares: mean. Further details on statistics are provided in the results and statistical summary file. Source data are provided as a Source Data file.

Supplementary Figure 8

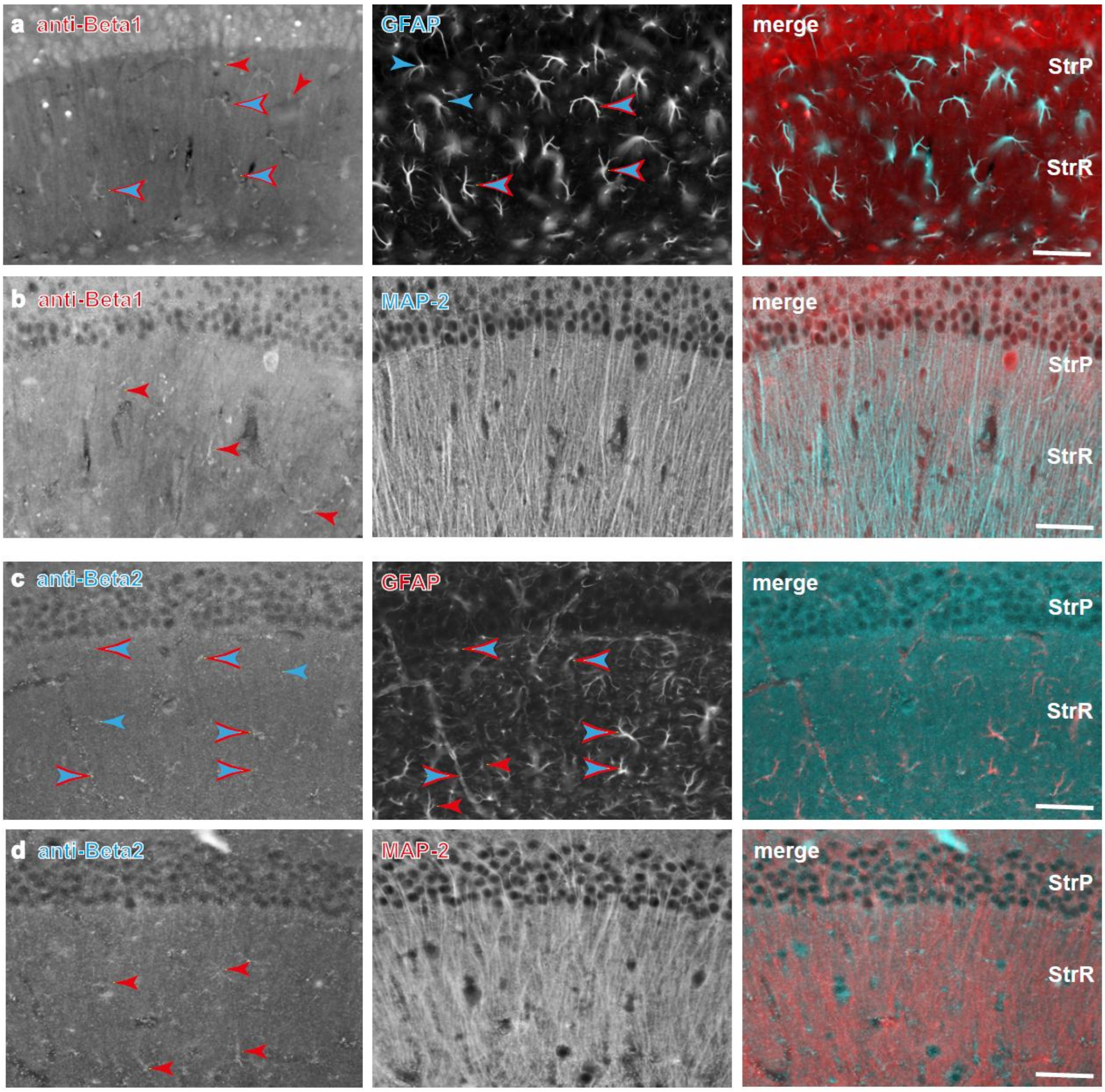

Suppl. Fig. 8: Spatial distribution of the NKA subunits β1 and β2 in GFAP- and MAP-2-positive cells of the CA1 region. a Co-expression of β1 and GFAP. Besides a somatic neuronal/perineuronal labelling for β1, GFAP-positive structures in the *stratum radiatum* label for β1, including apparent astrocyte somata and processes. Moreover, a small proportion of cells are positive for either β1 or GFAP. **b** Double label for β1 and MAP-2. β1 labelling is found at the neuronal/perineuronal surface of MAP-2-expressing cells as well as in proximal dendrites. In the *stratum radiatum*, labeling of structures with typical astrocytic morphology can be found. **c** Co-expression of β2 and GFAP. Besides a neuronal/perineuronal labelling for β2, the vast majority of GFAP-positive cell somata express β1. Moreover, proximal GFAP-positive processes show β2 labeling. Again, small subsets of cells expressed either β1 or GFAP. **d** Double label for β1 and MAP-2. Again, β1 labelling is found at the neuronal surface (and/or perineuronal) of MAP-2-expressing cells. Analogous to β1 staining, β2-positive, but MAP-2 negative somata as well as processes with a morphology typical for astrocytes are detectable. In all panels, monochromatic arrowheads point out cells exhibiting only one signal, double-colored arrowheads show cells labeled by both markers used. StrP: *stratum pyramidale*, StrR: *stratum radiatum*. Similar results were independently obtained at least 15 times for each experimental condition. Scale: 80 µm.

Supplementary Figure 9

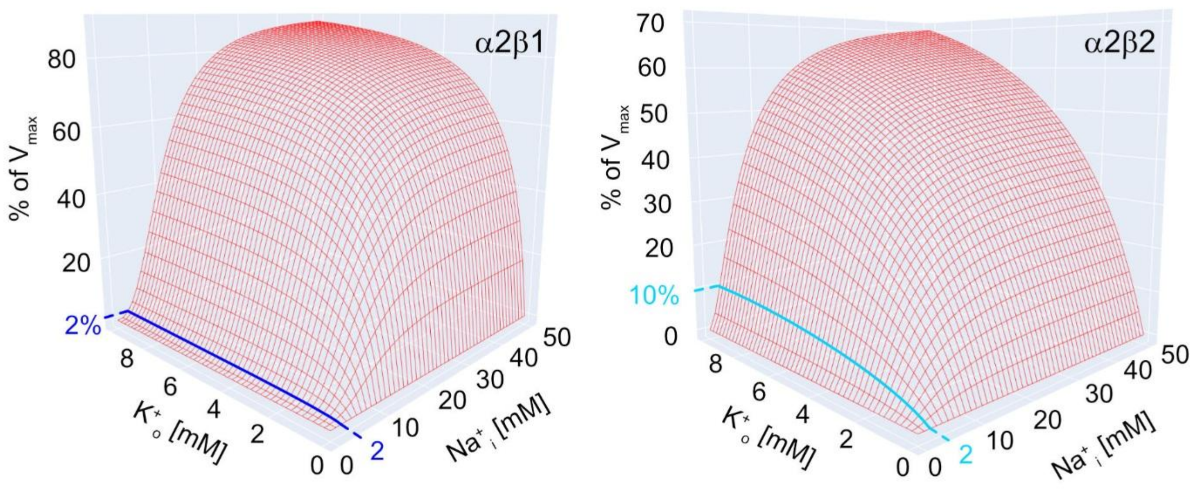

Suppl. Fig. 9: Differential activity profiles of the two isoforms of NKA as functions of intracellular Na^+ and extracellular K^+. The wireframe plots show the activity of NKA, normalized to their peak values (V_{max}) as we change $[Na^+]_i$ and $[K^+]_e$ for α2β1 (left) and α2β2 (right). Suppl. Table 6 lists the corresponding values for Hill's coefficients and affinities of both isoforms for $[Na^+]_i$ and $[K^+]_e$. The blue and turquoise lines highlight the dependency of NKA activity on intracellular Na^+. Note that when $[Na^+]_i$ drops to 2 mM, NKA activity is reduced to about 2% of V_{max} for α2β1 and to about 10% of V_{max} for α2β2, respectively.

Supplementary Note 1

Model equations for the dynamics of membrane potential and various ion concentrations in the astrocyte and extracellular space

The astrocyte model is adapted from (2-5) and focuses on the dynamics of various ionic channels, transporters, and cotransporters involved in astrocytic function. The Na^+/K^+-ATPase (NKA) maintains ionic homeostasis by actively exporting three Na^+ ions in exchange for two K^+ ions at the cost of ATP. In astrocytes, it works alongside other critical transporters to regulate ion dynamics and pH. The electrogenic Na^+-HCO_3^- cotransporter 1 (NBCe1) contributes by co-transporting Na^+ and HCO_3^- into the cell, aiding in intracellular pH regulation and influencing Na^+ accumulation. The Na^+/H^+-exchanger (NHE) further modulates pH and Na^+ levels by importing Na^+ while exporting protons. The model also includes glutamate transporters that mediate the uptake of glutamate into astrocytes, facilitating the recycling of neurotransmitters and preventing excitotoxicity. To regulate intracellular Ca^{2+}, Na^+/Ca^{2+}-exchangers (NCX) and Transient Receptor Potential Vanilloid 4 channels (TRPV4) are included. In forward mode, NCX removes excess Ca^{2+} while importing Na^+, and TRPV4 contributes to Ca^{2+} influx.

The model also incorporates Ca^{2+} exchange between the cytoplasm and the endoplasmic reticulum (ER) through sarcoplasmic/ER Ca^{2+}-ATPase (SERCA), inositol 1,4,5 trisphosphate (IP_3) receptors (IP_3Rs), and Ca^{2+} leak channels. In addition, the $Na^+/K^+/2Cl^-$ cotransporter 1 (NKCC1) and the K^+/Cl^- cotransporter 1 (KCC1), which are integral to the regulation of ionic balance, are included in the model. NKCC1 mediates the concurrent uptake of Na^+, K^+, and Cl^- from the extracellular space, contributing to the maintenance of intracellular ion concentrations, especially during conditions of elevated extracellular K^+. Conversely, KCC1 exports K^+ and Cl^- ions from the cell, playing a crucial role in the regulation of osmotic balance and volume recovery. Together, these transporters enable astrocytes to maintain their homeostatic functions while responding to neuronal activity.

All the above considerations lead to the following rate equations for the dynamics of membrane potential (V) and ion concentrations in the extra- and intracellular spaces in the ith compartment denoted by superscript i. Electrical coupling between neighboring compartments is represented by a coupling current (I_{Coup}) given by (6),

$$I_{\text{Coup}}^{(i)} = \sum_j \gamma_j^{(i)}\left(V^{(j)} - V^{(i)}\right). \quad [1]$$

If compartment i has length $L^{(i)}$ and radius $r^{(i)}$, and compartment j has length $L^{(j)}$ and radius $r^{(j)}$, then the inter-compartmental resistance is computed as the sum of the axial resistance from the midpoint of each compartment to their shared junction i.e.

$$R = \frac{R_a L^{(i)}}{2\pi\left(r^{(i)}\right)^2} + \frac{R_a L^{(j)}}{2\pi\left(r^{(j)}\right)^2} \quad [2]$$

where $R_a = 400\ \Omega cm^2$ is the intracellular resistivity. Inter-compartmental conductance, γ, is given by the inverse of the inter-compartmental resistance, divided by the total area of compartment i. The intracellular Na^+ and extracellular K^+ diffusion between coupled compartments are given by,

$$I_{DiffNa}^{(i)} = \text{intraD}_{\text{Na+}} \frac{A_{cs}^{(i)}}{L^{(i)}Vol^{(i)}} \sum_j \left([Na+]_\text{i}^{(j)} - [Na+]_\text{i}^{(i)} \right) \quad [3]$$

and $$I_{DiffK}^{(i)} = D_{\text{K+}} \frac{A_{\text{cs}}^{(i)}}{L^{(i)}\text{Vol}^{(i)}} \sum_j \left([\text{K}+]_\text{e}^{(j)} - [\text{K}+]_\text{e}^{(i)} \right). \quad [4]$$

Here, $D_{K+} = 250\,\mu m^2/s$ and $intraD_{Na+} = 600\,\mu m^2/s$ are the diffusion coefficients of K^+ and Na^+ (6), respectively. $A_{cs}^{(i)}$ and $Vol^{(i)}$ are cross-sectional area and volume of ith compartment, respectively.

All currents are in $\mu M/s$, and description of all symbols and their values are given in Suppl. Table 6. The initial values of different variables are given in Suppl. Table 7.

$$\frac{dV^{(i)}}{dt} = \text{conv}\Big(-I_{\text{NaLeak}}^{(i)} - I_{\text{KLeak}}^{(i)} - I_{\text{ClLeak}}^{(i)} - I_{\text{pump}}^{(i)} + I_{\text{NBCe1}}^{(i)} - I_{\text{NHE}}^{(i)} - 2I_{\text{TRPV}}^{(i)} - I_{\text{NCX}}^{(i)} + 2I_{\text{Glut}}^{(i)} + I_{\text{Coup}}^{(i)} \Big) \quad [5]$$

In the following, $g_{pathway}$ represents the maximum conductance of the respective ion pathway, and $E_{ion/pathway}$ the corresponding reversal potential. Here, the subscript *i* and *o* represent concentration in the intra- and extracellular space, respectively. Extracellular $[K^+]$ is represented by $[K^+]_e$.

$$I_{\text{NaLeak}}^{(i)} = g_{\text{NaLeak}}\left(V^{(i)} - E_{\text{Na}}\right); \quad [6]$$

$$g_{\text{Na}} = 293\ \mu\text{M/mV} \cdot \text{s}, \quad [7]$$

$$E_{\text{Na}} = 26.64 \ln\left(\frac{[\text{Na}^+]_\text{o}}{[\text{Na}^+]_\text{i}}\right). \quad [8]$$

$$I_{\text{KLeak}}^{(i)} = g_{\text{KLeak}}\left(V^{(i)} - E_K\right);. \quad [9]$$

$$g_{\text{K}} = 1243\ \mu\text{M/mV} \cdot \text{s},. \quad [10]$$

$$E_{\text{K}} = 26.64 \ln\left(\frac{[\text{K}^+]_\text{e}}{[\text{K}^+]_\text{i}}\right). \quad [11]$$

$$I_{\text{ClLeak}}^{(i)} = g_{\text{ClLeak}}\left(V^{(i)} - E_{\text{Cl}}\right); \quad [12]$$

$$g_{\text{Cl}} = 152\ \mu\text{M/mV} \cdot \text{s} \quad [13]$$

$$E_{\text{Cl}} = 26.64 \ln\left(\frac{[\text{Cl}^-]_i}{[Cl^-]_o}\right) \quad [14]$$

$$I_{\text{NBCe1}}^{(i)} = g_{\text{NBCe1}}\left(V^{(i)} - E_{\text{NBCe1}}\right); \quad [15]$$

$$g_{\text{NBCe1}} = 131\,\mu\text{M/mV} \cdot \text{s}. \quad [16]$$

$$E_{\text{NBCe1}} = -26.64 \ln\left(\frac{[\text{Na}^+]_\text{o}[\text{HCO}_3^-]_\text{o}^2}{[\text{Na}^+]_\text{i}[\text{HCO}_3^-]_\text{i}^2}\right). \quad [17]$$

$$I_{\text{NHE}}^{(i)} = g_{\text{NHE}}\left(V^{(i)} - E_{\text{NHE}}\right) \quad [18]$$

$$g_{\text{NHE}} = 131\ \mu\text{M/mV} \cdot \text{s} \quad [19]$$

$$E_{\mathrm{NHE}} = 26.64 \ln\left(\frac{[\mathrm{Na}^+]_\mathrm{o}[\mathrm{H}^+]_\mathrm{o}}{[\mathrm{Na}^+]_\mathrm{i}[\mathrm{H}^+]_\mathrm{i}}\right). \quad [20]$$

$$I_{\mathrm{TRPV4}}^{(i)} = g_{\mathrm{TRPV4}} \cdot m^{(i)} \cdot \left(V^{(i)} - E_{\mathrm{TRPV4}}\right). \quad [21]$$

m is the open probability of the TRPV4 channel, and

$$g_{\mathrm{TRPV4}} = 0.000317\ \mu\mathrm{M/mV} \cdot \mathrm{s}, \quad [22]$$

$$E_{\mathrm{TRPV4}} = 0.5 \ * \ 26.64 \ln\left(\frac{[\mathrm{Ca}^{2+}]_\mathrm{p}}{[\mathrm{Ca}^{2+}]_\mathrm{i}}\right). \quad [23]$$

Where $[Ca^{2+}]_p$ is the perivascular Ca^{2+} concentration, and the gating variable (m) for TRPV4 channel given by the following set of equations.

$$\frac{dm^{(i)}}{dt} = \frac{m_{\infty_i}^{(i)} - m^{(i)}}{t_{\mathrm{TRPV4}}}, \quad [24]$$

$$m_{\infty_i}^{(i)} = \frac{1.0}{1.0+\exp\left(\frac{-(\eta-\eta_O)}{\kappa}\right)} \cdot \left(\frac{1.0}{1.0+\mathrm{HCa_i}}\right) \cdot \left(\mathrm{HCa_i} + \tanh\left(\frac{V^{(i)} - v_{1\mathrm{TRPV4}}}{v_{2\mathrm{TRPV4}}}\right)\right), \quad [25]$$

$$\mathrm{HCa_i} = \frac{[\mathrm{Ca}^{2+}]_i}{\gamma_{\mathrm{Cai}}} + \frac{[\mathrm{Ca}^{2+}]_p}{\gamma_{\mathrm{Cao}}}, \quad [26]$$

$$\eta = \frac{R - R_{0,\mathrm{passive}}}{R_{0,\mathrm{passive}}}. \quad [27]$$

The fluxes through NCX and glutamate transporters are modeled as

$$I_{\mathrm{NCX}}^{(i)} = I_{\mathrm{NCXmax}} \cdot \frac{[\mathrm{Na}^+]_o^3}{\mathrm{K}_{\mathrm{NCXmN}}^3 + [\mathrm{Na}^+]_o^3} \cdot \frac{[\mathrm{Ca}^{2+}]_o}{\mathrm{K}_{\mathrm{NCXmC}} + [\mathrm{Ca}^{2+}]_o} \cdot \frac{\left(\frac{[\mathrm{Na}^+]_i^3}{[\mathrm{Na}^+]_o^3} e^{\eta_{\mathrm{NCX}} V^{(i)}/26.6} - \frac{[\mathrm{Ca}^{2+}]_i}{[\mathrm{Ca}^{2+}]_o} e^{(\eta_{\mathrm{NCX}}-1)V^{(i)}/26.6}\right)}{1 + k_{\mathrm{sat}} e^{(\eta_{\mathrm{NCX}}-1)V^{(i)}/26.6}} \cdot \frac{1000\mathrm{A}^{(\mathrm{i})}}{F \cdot \mathrm{Vol}^{(\mathrm{i})}}; \quad [28]$$

$$I_{\mathrm{GluT}}^{(i)} = I_{\mathrm{GluTmax}} \cdot \frac{[\mathrm{K}^+]_\mathrm{i}}{[\mathrm{K}^+]_\mathrm{i} + K_{\mathrm{GluTmK}}} \cdot \frac{[\mathrm{Na}^+]_\mathrm{o}^3}{[\mathrm{Na}^+]_\mathrm{o}^3 + K_{\mathrm{GluTmN}}^3} \cdot \frac{[\mathrm{Glu}]}{[\mathrm{Glu}] + K_{GluTmg}} \cdot \frac{1000\mathrm{A}^{(\mathrm{i})}}{F \cdot \mathrm{Vol}^{(\mathrm{i})}}. \quad [29]$$

Where $\frac{1000\mathrm{A}^{(\mathrm{i})}}{F \cdot \mathrm{Vol}^{(\mathrm{i})}}$ converts mS/cm^2 to $\frac{\mu M}{s}$ and $A^{(i)}$ is the surface area of the compartment in m^2 and $Vol^{(i)}$ is its volume in m^3.

For a point model, $A^{(i)} = A_c$ [30]

and $\mathrm{Vol}^{(i)} = \mathrm{Vol_c}$ [31]

So, $\mathrm{A_c} = 4\pi \left(\frac{3\,\mathrm{Vol_c}}{4\pi}\right)^{\frac{2}{3}}$. [32]

The concentrations of various ions are modeled with the following equations:

$$\frac{d[\mathrm{K}^+]_i^{(i)}}{dt} = -I_{K,\mathrm{leak}}^{(i)} + 2 \cdot I_{\mathrm{pump}}^{(i)} + I_{\mathrm{NKCC1}}^{(i)} + I_{\mathrm{KCC1}}^{(i)} - I_{\mathrm{GluT}}^{(i)}, \quad [33]$$

$$\frac{d[\mathrm{Na}^+]_\mathrm{i}^{(i)}}{dt} = -I_{\mathrm{Na,leak}}^{(i)} - 3 \cdot I_{\mathrm{pump}}^{(i)} + I_{\mathrm{NBC}}^{(i)} - I_{\mathrm{NHE}}^{(i)} + I_{\mathrm{NKCC1}}^{(i)} + I_{\mathrm{DiffNa}}^{(i)} - 3 \cdot I_{\mathrm{NCX}}^{(i)} + 3 \cdot I_{\mathrm{GluT}}^{(i)}, \quad [34]$$

$$\frac{d[\mathrm{Ca}^{2+}]_i^{(i)}}{dt} = B_{\mathrm{cyt}}\left(I_{\mathrm{NCX}}^{(i)} + I_{\mathrm{IP3}}^{(i)} - I_{\mathrm{spump}}^{(i)} + I_{\mathrm{ERleak}}^{(i)} + \frac{I_{\mathrm{TRPV}}^{(i)}}{r_{\mathrm{buff}}}\right), \quad [35]$$

$$\frac{d[\mathrm{Cl}^-]_i^{(i)}}{dt} = \frac{d[\mathrm{Na}^+]_i^{(i)}}{dt} + \frac{d[\mathrm{K}^+]_\mathrm{i}^{(i)}}{dt} + 2 \cdot \frac{d[\mathrm{Ca}^{2+}]_\mathrm{i}^{(i)}}{dt}, \quad [36]$$

$$\frac{d[\mathrm{K}^+]_\mathrm{e}^{(i)}}{dt} = VR\left(I_{K,\mathrm{leak}}^{(i)} - 2 \cdot I_{\mathrm{pump}}^{(i)} - I_{\mathrm{NKCC1}}^{(i)} - I_{\mathrm{KCC1}}^{(i)} + I_{\mathrm{GluT}}^{(i)}\right) + I_{\mathrm{DiffK}}^{(i)} + I_{\mathrm{Kbath\_to\_Ke}}^{(i)}, \quad [37]$$

$$\frac{d[\mathrm{Na}^+]_\mathrm{o}^{(i)}}{dt} = VR\left(I_{\mathrm{Na,leak}}^{(i)} + 3 \cdot I_{\mathrm{pump}}^{(i)} - I_{\mathrm{NBC}}^{(i)} + I_{\mathrm{NHE}}^{(i)} - I_{\mathrm{NKCC1}}^{(i)} + 3 \cdot I_{\mathrm{NCX}}^{(i)} - 3 \cdot I_{\mathrm{GluT}}^{(i)}\right), \quad [38]$$

$$\frac{dpH_i^{(i)}}{dt} = \frac{I_{\mathrm{NHE}}^{(i)} + 2 \cdot I_{\mathrm{NBC}}^{(i)}}{\beta_i}, \quad [39]$$

$$\frac{dpH_o^{(i)}}{dt} = VR\left(\frac{-I_{\mathrm{NHE}}^{(i)} - 2 \cdot I_{\mathrm{NBC}}^{(i)}}{\beta_o}\right) + 0.5 \cdot \left(7.35 - \mathrm{pH}_\mathrm{i}^{(i)}\right). \quad [40]$$

The extra- and intracellular bicarbonate and proton concentrations are given as

$$[\mathrm{HCO_3}]_\mathrm{o}^{(i)} = 10^{\left(\mathrm{pH}_\mathrm{o}^{(i)} - pK_a\right)} \cdot \mathrm{s} \cdot \left[P_{CO_2}\right], \quad [41]$$

$$[\mathrm{HCO_3}]_\mathrm{i}^{(i)} = 10^{\left(pH_i^{(i)} - pH_o^{(i)}\right)} \cdot [\mathrm{HCO_3}]_\mathrm{o}^{(i)}, \quad [42]$$

$$\left[\mathrm{H}^+\right]_\mathrm{o}^{(i)} = \frac{s \cdot k_h \cdot \left[P_{\mathrm{CO_2}}\right]}{[\mathrm{HCO_3^-}]_\mathrm{o}^{(i)}}, \quad [43]$$

$$\left[\mathrm{H}^+\right]_\mathrm{i}^{(i)} = \frac{s \cdot k_h \cdot \left[P_{\mathrm{CO_2}}\right]}{[\mathrm{HCO_3^-}]_\mathrm{i}^{(i)}}, \quad [44]$$

The intrinsic intra- and extracellular cellular buffering capacity of bicarbonate, and intracellular Ca^{2+} are modeled as

$$\beta_i^{(i)} = 25000 + 2.3 \cdot [\mathrm{HCO_3^-}]_\mathrm{i}^{(i)}, \quad [45]$$

$$\beta_o^{(i)} = 10000 + 2.3 \cdot [\mathrm{HCO_3^-}]_\mathrm{o}^{(i)}. \quad [46]$$

$$B_{\mathrm{cyt}} = \left(1 + \mathrm{BK_{end}} + \frac{K_{ex} \cdot B_{ex}}{\left(K_{ex} + [\mathrm{Ca}^{2+}]_\mathrm{i}\right)^2}\right)^{-1}. \quad [47]$$

The flux through $\alpha_2\beta_1$ and $\alpha_2\beta_2$ isoforms of NKA are modeled by using the previously reported values for their binding affinities for intracellular Na^+ and extracellular K^+.

$$I_{\mathrm{pump\_\alpha_2\beta_1}}^{(i)} = I_{\mathrm{pump\_max}} \cdot \frac{[\mathrm{Na}^+]_\mathrm{i}^{\mathrm{HC}_{\alpha_2\beta_1}}}{[\mathrm{Na}^+]_\mathrm{i}^{\mathrm{HC}_{\alpha_2\beta_1}} + K_{\mathrm{NaK}_{\alpha_2\beta_1}}^{\mathrm{HC}_{\alpha_2\beta_1}}} \cdot \frac{[\mathrm{K}^+]_\mathrm{e}}{[\mathrm{K}^+]_\mathrm{e} + K_{\mathrm{Ks}_{\alpha_2\beta_1}}}, \quad [48]$$

$$I_{\mathrm{pump\_\alpha_2\beta_2}}^{(i)} = I_{\mathrm{pump\_max}} \cdot \frac{[\mathrm{Na}^+]_\mathrm{i}^{\mathrm{HC}_{\alpha_2\beta_2}}}{[\mathrm{Na}^+]_\mathrm{i}^{\mathrm{HC}_{\alpha_2\beta_2}} + K_{\mathrm{NaK}_{\alpha_2\beta_2}}^{\mathrm{HC}_{\alpha_2\beta_2}}} \cdot \frac{[\mathrm{K}^+]_\mathrm{e}}{[\mathrm{K}^+]_\mathrm{e} + K_{\mathrm{Ks}_{\alpha_2\beta_2}}}. \quad [49]$$

These two fluxes, normalized to their peak values, were fitted to the data in Ref. (7, 9) and are shown in Suppl. Fig. 9 and (Link).

The activity of NKCC1 and KCC1, and the diffusion of K^+ from bath solution to extracellular space are given as

$$I_{\mathrm{NKCC1}}^{(i)} = 26.7 g_{\mathrm{NKCC1}} \cdot \ln\left(\frac{[\mathrm{Na^+}]_{\mathrm{o}} \cdot [\mathrm{K^+}]_{\mathrm{e}} \cdot [\mathrm{Cl^-}]_{\mathrm{o}}^2}{[\mathrm{Na^+}]_{\mathrm{i}} \cdot [\mathrm{K^+}]_{\mathrm{i}} \cdot [\mathrm{Cl^-}]_{\mathrm{i}}^2}\right), \quad [50]$$

$$I_{\mathrm{KCC1}}^{(i)} = 26.7 g_{\mathrm{KCC1}} \cdot \ln\left(\frac{[\mathrm{K^+}]_{\mathrm{e}} \cdot [\mathrm{Cl^-}]_{\mathrm{o}}}{[\mathrm{K^+}]_{\mathrm{i}} \cdot [\mathrm{Cl^-}]_{\mathrm{i}}}\right). \quad [51]$$

$$I_{\mathrm{Kbath\_to\_Ke}}^{(i)} = 50.0 \cdot ([\mathrm{K_{bath}^+}] - [\mathrm{K_e^+}]). \quad [52]$$

Ca^{2+} fluxes through pathways in the ER membrane (IP_3R, SERCA, and leak) are modeled as

$$I_{\mathrm{IP3}}^{(i)} = I_{\mathrm{IP3max}} \cdot \left(\frac{[\mathrm{IP3}]_{\mathrm{i}}^{(i)}}{[\mathrm{IP3}]_{\mathrm{i}}^{(i)} + K_I} \cdot \frac{[\mathrm{Ca^{2+}}]_{\mathrm{i}}^{(i)}}{[\mathrm{Ca^{2+}}]_{\mathrm{i}}^{(i)} + K_{\mathrm{act}}} \cdot h_{\mathrm{IP3}}^{(i)}\right)^3 \cdot \left(1.0 - \frac{[\mathrm{Ca^{2+}}]_{\mathrm{i}}^{(i)}}{[\mathrm{Ca^{2+}}]_{\mathrm{ER}}}\right), \quad [53]$$

$$I_{\mathrm{spump}}^{(i)} = V_{max} \cdot \frac{[Ca^{2+}]_i^2}{[Ca^{2+}]_i^2 + k_{pump}^2}, \quad [54]$$

$$I_{\mathrm{ERleak}}^{(i)} = P_L \cdot \left(1.0 - \frac{[\mathrm{Ca^{2+}}]_{\mathrm{i}}}{[\mathrm{Ca^{2+}}]_{\mathrm{ER}}}\right). \quad [55]$$

Where the IP_3 concentration depends on the production due to glutamate (G) and decay over time.

$$\frac{d[\mathrm{Ip_3}]^{(i)}}{dt} = \rho_h \cdot G - k_{deg} \cdot [Ip_3]^{(i)}, \quad [56]$$

$$\text{with } G = \frac{\rho + \delta}{K_G + \rho + \delta}, \quad [57]$$

$$\rho = \rho_{min} + \frac{\rho_{max} - \rho_{min}}{Glu_{\mathrm{max}}} \cdot \mathrm{Glu}. \quad [58]$$

Simulating the spatial profile of intracellular Na^+

To simulate the spatial profile of intracellular Na^+, we make the Na^+ influx through EAAT and the relative expression of NKA isoforms throughout the cell as functions of distance from the soma. To do this, we calculate the maximum distance along each branch and calculate the relative distance (R) of each compartment with respect to soma, where R is 0 at the soma and 1 at the terminal of the branch. This ratio is then used to distribute NKA pumps along the branch. For example, the compartment closer to soma will predominantly express α2β1. As we move farther from soma, α2β2 becomes dominant, and the pump current at each compartment is calculated accordingly. Accordingly, the pump expression at a compartment with relative distance R is given by

$$I_{pump} = (1 - R) \times I_{\mathrm{pump\_\alpha2\beta1}} + R \times I_{\mathrm{pump\_\alpha2\beta2}}. \quad [59]$$

The same logic is applied to increase the maximum glutamate transport via EAAT1. It starts at 100% in the soma and increases linearly with distance to 300% at the branch terminal (0.1 pA/μm^2 to 0.3 pA/μm^2) using the following relation:

$$I_{\mathrm{GluTmax}} = 0.1 + 0.2 \times R. \qquad [60]$$

This value is then used to calculate glutamate flux at each compartment. In simulation where I_{GluTmax} is varied, we fix the subunit ratio of NKA at 30% of $\alpha_2\beta_1$ and 70% of $\alpha_2\beta_2$ (9-11).

Supplementary Table 1: Primary Antibodies Used

Antibody	Manufacturer	Order-No.	Lot No.	Dilution	Identifier	Type	Host
Anti-Beta 2 Na^+/K^+ ATPase	Alomone	ANP-012	ANP012AN0225	1:100	RRID:AB_2756682	polyclonal	rabbit
Anti-atp1b2a Antibody	MyBiosource	MBS648563	L22011859	1:50	TBD	polyclonal	rabbit
Anti-ATP1b1 Antibody	MyBiosource	MBS2077952	A20251005788 A20251128321	1:10	TBD	polyclonal	rabbit
Anti-MAP2 antibody [HM-2]	abcam	ab11267	1093223-1	1:500	RRID: AB_297885	monoclonal	mouse
Monoclonal Anti-Glial Fibrillary Acidic Protein (GFAP) antibody	Sigma	G3893-100UL	096M4844V	1:500	RRID: AB_477010	monoclonal	mouse
Polyclonal Rabbit Anti-Glial Fibrillary Acidic Protein	Agilent	Z0334	20071831	1:500	RRID: AB_10013382	polyclonal	rabbit

Supplementary Table 2: Secondary Antibodies Used (diluted 1:100)

Antibody	Manufacturer	Order-No.	Lot No.	Identifier	Host
Alexa Fluor® PLUS 488 goat anti-rabbit IgG (H+L)	Thermo Fisher	A32731TR	AB412023	RRID:AB_286649	goat
Alexa Fluor® PLUS 405 goat anti-rabbit IgG (H+L)	Thermo Fisher	A48254	ZE396879	RRID:AB_2890548	goat
AlexaFluor®594, goat anti-mouse IgG (H+L)	Thermo Fisher	A11005	2897811	RRID:AB_2534073	goat
CF®488A Donkey Anti-Rabbit IgG (H+L)	Biotium	20015	20c1117	RRID:AB_10559669	donkey

<u>**Supplementary Table 3**</u>**:**

Single- and double labelling for NKA subunits ß1 and ß2

**Single label Na^+/K^+-ATPase ß1**<br>n=6 animals and 4 sections/animal			**Single label Na^+/K^+-ATPase ß2**<br>n=6 animals and 5 sections/animal			**Double Label Na^+/K^+-ATPase ß1 and ß2**<br>n=5 animals and 3 sections/animal		
**Step**	**Compound**	**Duration/ Condition/ Dilution**	**Step**	**Compound**	**Duration/ Condition/ Dilution**	**Step**	**Compound**	**Duration/ Condition/ Dilution**
Blocking/ Permeabilization	PBS containing<br>0.25% TX<br>and 2% NGS	90 min<br>RT<br>1:10	Blocking	PBS<br>containing<br>2% NGS	90 min<br>RT<br>1:100	Blocking	PBS<br>containing<br>2% NGS	90 min<br>RT
Primary Antibody	rb anti-ATP1b1	Overnight<br>4 °C<br>1:10	Primary Antibody	rb anti-Beta 2<br>Na^+/K^+-ATPase<br>rb anti-atp1b2a	Overnight<br>4 °C<br>1:100/1:50	Primary Antibody	rb anti-Beta 2<br>Na^+/K^+-ATPase	Overnight<br>4 °C<br>1:100
Wash	PBS containing<br>0.25% TX<br>and 2% NGS	3 x 20 min<br>RT	Wash	PBS<br>containing<br>2% NGS	3 x 20 min<br>RT	Wash	PBS<br>containing<br>2% NGS	3 x 20 min<br>RT
Secondary Antibody	gt anti rb Alexa Fluor® PLUS 488<br>*or*<br>gt anti rb Alexa Fluor® PLUS 405	60 min<br>RT<br>1:100	Secondary Antibody	gt anti rb Alexa Fluor® PLUS 488<br>*or*<br>gt anti rb Alexa Fluor® PLUS 405	60 min<br>RT<br>1:100	Secondary Antibody	gt anti rb Alexa Fluor® PLUS 488<br>*or*<br>gt anti rb Alexa Fluor® PLUS 405	60 min<br>RT<br>1:100
Wash	PBS containing<br>0.25% TX<br>and 2% NGS	3 x 20 min<br>RT	Wash	PBS<br>containing<br>2% NGS	3 x 20 min<br>RT	Wash	PBS<br>containing<br>2% NGS	3 x 20 min<br>RT
Coverslipping	Mowiol/ DABCO		Coverslipping	Mowiol/ DABCO		Coverslipping	PBS	
Documentation			Documentation			Documentation		
						Bleaching		3 hrs
						Blocking/ Permeabilizat-ion	PBS containing<br>0.25% TX<br>and 2% NGS	90 min<br>RT
						Primary Antibody	rb anti-ATP1b1	Overnight<br>4 °C
						Wash	PBS containing<br>0.25% TX<br>and 2% NGS	3 x 20 min<br>RT<br>1:10
						Secondary Antibody	gt anti rb Alexa Fluor® PLUS 488<br>*or*<br>gt anti rb Alexa Fluor® PLUS 405	60 min<br>RT<br>1:100
						Wash	PBS containing<br>0.25% TX<br>and 2% NGS	3 x 20 min<br>RT
						Coverslipping	Mowiol/ DABCO	
						Documentation		

gt: Goat, mo: Mouse, NGS: Normal Goat Serum, PBS: Phosphate-Buffered Saline, rb: Rabbit, RT: Room Temperature, TX: Triton-X-100.

Negative controls were run in parallel to each staining by omitting either all or one of the primary antibodies. Per staining at least 2 sections for double-negative and 2 sections for each singe-negative control were processed.

Supplementary Table 4: Double-labelling for NKA subunits ß1/ß2 and GFAP/MAP2

**Double-label for Na^+/K^+-ATPase ß2 vs. GFAP/MAP2** n=5 animals and 4 sections/animal			**Double-label for Na^+/K^+-ATPase ß1 vs. GFAP/MAP-2** n=5 animals and 3 sections/animal		
**Step**	**Compound**	**Duration/ Condition/ Dilution**	**Step**	**Compound**	**Duration/ Condition/ Dilution**
Blocking	PBS containing 2% NGS	90 min RT	Blocking	PBS containing 0.25% TX and 2% NGS	90 min RT
Primary Antibody	rb anti-Beta 2 Na^+/K^+-ATPase rb anti-atp1b2a	Overnight 4 °C 1:100/1:50	Primary Antibody	rb anti-ATP1b1	Overnight 4 °C 1:10
Wash	PBS containing 2% NGS	3 x 20 min RT	Wash	PBS containing 0.25% TX and 2% NGS	3 x 20 min RT
Secondary Antibody	gt anti rb Alexa Fluor® PLUS 488 *or* gt anti rb Alexa Fluor PLUS 405	60 min RT	Secondary Antibody	gt anti rb Alexa Fluor® PLUS 488 *or* gt anti rb Alexa Fluor PLUS 405	60 min RT
Wash	PBS containing 2% NGS	3 x 20 min RT	Wash	PBS containing 0.25% TX and 2% NGS	3 x 20 min RT
Blocking/ Permeabilizatiion	PBS containing 0.25% TX and 2% NGS	90 min RT	Blocking/ Permeabilization	PBS containing 0.25% TX and 2% NGS	90 min RT
Primary Antibody	mo anti GFAP *or* mo anti MAP-2	Overnight 4 °C 1:500	Primary Antibody	mo anti GFAP *or* mo anti MAP-2	Overnight 4 °C 1:1000
Wash	PBS containing 0.25% TX and 2% NGS	3 x 20 min RT	Wash	PBS containing 0.25% TX and 2% NGS	3 x 20 min RT
Secondary Antibody	with gt anti mo AlexaFluor 594	60 min RT 1:100	Secondary Antibody	with gt anti mo AlexaFluor 594	60 min RT 1:100
Wash	PBS containing 0.25% TX and 2% NGS	3 x 20 min RT	Wash	PBS containing 0.25% TX and 2% NGS	3 x 20 min RT
Coverslipping	Mowiol/ DABCO		Coverslipping	Mowiol/ DABCO	
Documentation			Documentation		

GFAP: Glial Fibrillary acidic Protein, gt: Goat, MAP_2 Microtubule-Associated Protein-2, mo: Mouse.
NGS: Normal Goat Serum, PBS: Phosphate-Buffered Saline, rb: Rabbit, RT: Room Temperature, TX: Triton-X-100.

Negative controls were run in parallel to each staining by omitting either all or one of the primary antibodies.

Supplementary Table 5:

Simulation of the influence of subunit composition and/or expression of NKA together with the strength of Na^+ influx on intracellular [Na^+] of astrocytes

Composition	NKA strength	Na^+ [mM] 60% Na^+ influx	Na^+ [mM] 100% Na^+ influx	Na^+ [mM] 170% Na^+ influx
**100% α2β1**	100%	8.71	11.79	18.61
**100% α2β2**	100%	10.00	22.40	62.00
**30% α2β1 / 70% α2β2**	60%	18.95	52.24	95.99
**30% α2β1 / 70% α2β2**	180%	5.52	7.96	12.47

Supplementary Table 6:

Description and values of various parameters used in the model

Symbol	Description	Value
conv	Scaling factor to convert µM/s to mV/s	1970 mV/µM
I_{NCXmax}	Maximum current through NCX	0.001 pA/µM^2
K_{NCXmN}	Half-saturation constant for Na^+	87500 µM
K_{NCXmC}	Half-saturation constant for Ca^{2+}	1380 µM
K_{sat}	Saturation factor ensuring saturation at large negative potentials	0.1
η_{NCX}	Energy barrier position	0.35
F	Faraday constant	96500 C/mol
Vol_c	Cell volume	168 µm^3
$I_{GluTmax}$	Maximum current through glutamate transporter	0.75 pA/µM^2
K_{GluTmK}	K^+ binding rate to glutamate transporter	5000 µM
K_{GluTmN}	Na^+ binding rate to glutamate transporter	15000 µM
K_{GluTmg}	Glutamate binding rate to glutamate transporter	34 µM
r_{buff}	Rate of Ca^{2+} buffering at the endfeet compared to the astrocyte body	0.05
VR	Volume ratio between the compartment and the extracellular space	0.33
pK_a	Negative logarithm of the acid dissociation constant of carbonic acid	6.1
s	Product of solubility in aqueous solution	0.225 µM/Pa
$[P_{CO_2}]$	Partial pressure of carbon dioxide	5332.9 Pa
k_h	Dissociation constant of carbonic acid	0.8 µM
I_{IP3max}	Maximum IP_3 current	2880 µM/s
K_I	Disassociation constant for IP_3 binding to an IP_3R	0.03 µM
K_{act}	Disassociation constant of Ca^{2+} binding to the activation site of IP_3-receptor	0.17 µM
V_{max}	Maximum rate of SERCA	20 µM/s
k_{pump}	SERCA dissociation constant	0.24 µM
P_{L}	ER leak channel steady-state balance constant	0.0804 µM/s

ρ_h	Maximum rate of IP_3 production in astrocytes due to glutamate receptors	8.6 μM/s
k_{deg}	Rate constant for IP_3 degradation in astrocyte	1.25 /s
t_{TRPV4}	Characteristic time constant for m	0.9
R	Vessel radius	23 µm
$R_{0,\text{passive}}$	Vessel radius when passive (no strain applied)	20 µm
η_{o}	Strain required for half activation of the TRPV4 channel	0.1
κ	TRPV4 channel strain constant	0.1
γ_{Cai}	Intracellular Ca^{2+} concentration constant	200 µM
γ_{Cao}	Extracellular Ca^{2+} concentration constant	0.01 µM
v_{1TRPV4}	TRPV4 channel voltage gating constant	120 mV
v_{2TRPV4}	TRPV4 channel voltage gating constant	13 mV
BK_{end}	Ratio of endogenous buffer concentration to dissociation constant	40
K_{ex}	Dissociation constant of exogenous buffer	0.26 µM
B_{ex}	Concentration of exogenous buffer	11.35 µM
ρ_{min}	Minimum ratio of bound to unbound IP_3 receptors	0.1
ρ_{max}	Maximum ratio of bound to unbound IP_3 receptors	0.7
KG	G-protein dissociation constant	8.82
δ	Ratio of the activities of the unbound and bound receptors	0.01235
Glu_{max}	Maximum glutamate concentration	1846 µM
I_{pump_max}	Maximum current through NKA	35.5 µM/s
$HC_{\alpha_2\beta_1}$	Na^+ Hill's coefficient for $\alpha_2\beta_1$ NKA isoform	2.39
$HC_{\alpha_2\beta_2}$	Na^+ Hill's coefficient for $\alpha_2\beta_2$ NKA isoform	1.55
$K_{\text{NaK}_{\alpha_2\beta_1}}$	Half-saturation constant for intracellular Na^+ binding to NKA ($\alpha_2\beta_1$)	10600 µM
$K_{\text{NaK}_{\alpha_2\beta_2}}$	Half-saturation constant for intracellular Na^+ binding to NKA ($\alpha_2\beta_2$)	6800 µM
$K_{Ks_{\alpha_2\beta_1}}$	Half-saturation constant for extracellular K^+ binding to NKA ($\alpha_2\beta_1$)	910 µM
$K_{Ks_{\alpha_2\beta_2}}$	Half-saturation constant for extracellular K^+ binding to NKA ($\alpha_2\beta_2$)	3600 µM

<u>Supplementary Table 7:</u>

Initial values of different variables

**Variable**	**Value**
$[Na^+]_i$	17000 µM
$[K^+]_i$	146000 µM
$[Ca^+]_i$	0.082 µM
$[Na^+]_o$	152000 µM
$[K^+]_e$	2900 µM
V	-80 mV
$[IP_3]_i$	0.0483 µM
pH_i	7.33
pH_o	7.35

References

1) Bhattarai A, Meyer J, Petersilie L, Shah SI, Neu LA, Rose CR, Ullah G (2024*): Deep-Learning-Based Segmentation of Cells and Analysis (DL-SCAN*). Biomolecules **14**: 1348. doi: 10.3390/biom14111348
2) Everaerts K, Thapaliya P, Pape N, Durry S, Eitelmann S, Roussa E, Ullah G & Rose CR (2023): *Inward Operation of Sodium-Bicarbonate Cotransporter 1 Promotes Astrocytic Na^+ Loading and Loss of ATP in Mouse Neocortex during Brief Chemical Ischemia*. Cells Nov 21; **12**(23):2675. doi: 10.3390/cells12232675.
3) Thapaliya P, Pape N, Rose CR & Ullah G (2023): *Modeling the heterogeneity of sodium and calcium homeostasis between cortical and hippocampal astrocytes and its impact on bioenergetics.* Front Cell Neurosci Jan 30; **17**:1035553. doi: 10.3389/fncel.2023.1035553.
4) Kenny A, Plank MJ & David T (2018): *The role of astrocytic calcium and TRPV4 channels in neurovascular coupling.* J Comput Neurosci, **44**(1): p. 97-114.
5) Oschmann F (2018): *Computational Modeling of Glutamate-Induced Calcium Signal Generation and Propagation in Astrocytes*. Dissertation, Technische Universitaet Berlin, Berlin, Germany.
6) Nelson JSE, Meyer J, Gerkau NJ, Kafitz KW, Ullah G, Santamaria F, Rose CR (2025): Spatio-temporal dynamics of lateral Na^+ diffusion in apical dendrites of mouse CA1 pyramidal neurons. J Neurosci, **45**(44):e0077252025. doi: 10.1523/JNEUROSCI.0077-25.2025.
7) Larsen BR, Stoica A, MacAulay N (2016a): *Managing Brain Extracellular K^+ during Neuronal Activity: The Physiological Role of the Na^+/K^+-ATPase Subunit Isoforms*. Frontiers in Physiology, **7**:141.
8) Larsen BR, Holm R, Vilsen B, MacAulay N (2016b): *Glutamate transporter activity promotes enhanced Na^+/K^+-ATPase-mediated extracellular K^+ management during neuronal activity*. J Physiol **594**:6627-6641.
9) Zhang Y, Chen K, Sloan SA, Bennett ML, Scholze AR, O'Keeffe S, Phatnani HP, Guarnieri P, Caneda C, Ruderisch N, Deng S, Liddelow SA, Zhang C, Daneman R, Maniatis T, Barres BA, Wu JQ (2014): *An RNA-Sequencing Transcriptome and Splicing Database of Glia, Neurons, and Vascular Cells of the Cerebral Cortex*. J Neurosci **34**:11929-11947.
10) Clarke LE, Liddelow SA, Chakraborty C, Munch AE, Heiman M, Barres BA (2018): *Normal aging induces A1-like astrocyte reactivity*. Proc Natl Acad Sci U S A **115**:E1896-E1905.
11) Batiuk MY, Martirosyan A, Wahis J, de Vin F, Marneffe C, Kusserow C, Koeppen J, Viana JF, Oliveira JF, Voet T, Ponting CP, Belgard TG, Holt MG (2020*): Identification of region-specific astrocyte subtypes at single cell resolution*. Nature Communications **11**:1220.
