# Supplementary material for: Cellular and subcellular heterogeneity of astrocytic Na⁺ homeostasis tuning astrocytes into functionally distinct subgroups in the mouse brain
Source: Nat Commun. 2026 May 20;17:4515. doi: 10.1038/s41467-026-73435-z (PMC13190688; doi:10.1038/s41467-026-73435-z)
Supplement: Supplementary file 2 — Description of Additional Supplementary Files [file 41467_2026_73435_MOESM2_ESM.pdf]

Description of Additional Supplementary Files

Supplemental Statistical Summary
