# Supplementary material for: Cellular and subcellular heterogeneity of astrocytic Na⁺ homeostasis tuning astrocytes into functionally distinct subgroups in the mouse brain
Source: Nat Commun. 2026 May 20;17:4515. doi: 10.1038/s41467-026-73435-z (PMC13190688; doi:10.1038/s41467-026-73435-z)
Supplement: Supplementary file 3 — Additional Supplementary File - Statistical Summary [file 41467_2026_73435_MOESM3_ESM.pdf]

Data	Mean	SD	Median	n	n_p	N	Statistical Test used	Paired/ unpaired								
**Baseline**																
Baseline Na *in situ* (Fig. 1f)	13,8	7,2	12,8	369		32	MWU	unpaired		1,70E-04 *** U=12246; Z=3.76		0,53294 n.s. MWU unpaired U=5868; Z=-0.62	0,85 n.s. MWU unpaired	0,00408 ** MWU unpaired U= 3485, Z=-2.87	3,80E-04 *** MWU unpaired U=6370; Z=-3.55	2,56E-06 *** MWU unpaired U=30850; Z=4.70
Baseline In vivo (Fig. 2g)	19,8	10,6	18,3	50		12	MWU	unpaired			0,00168 ** U=1195; Z=3.14					
Cortex (Fig. 2c)	12,5	4,2	12,0	34		4										
Baseline Na+ SBFI/Ratio (Suppl. Fig. 1e, f)	13,1	5,4	13,1	46		8	MWU	unpaired								
Baseline Subset (Fig 1i)	12.8	6.4	13.6	13		4	PS TT	paired	7.36E-06 *** DF=12							
0 Na Calibration Subset (Fig. 1j)	1,3	1,5	0,9	13		4										
0 Na Subset (Fig. 1j)	0,5	0,8	0,0	121		3	MWU	unpaired	0,26341 n.s. U=7838, Z=1.12							
0 Na Calibration Subset (Fig. 1j)	0,4	0,7	0,0	121		3										
**Processes**	**Mean**	**SD**	**Median**	**n**	**n_p**	**N**										
Processes average per cell (Fig. 3b)	17,3	6,5	17,1	29		29				0,66553 n.s. MWU unpaired U=682, Z=-0.43						
Processes subset (Fig. 3c)	17,0	3,6	17,1	7		7	PS TT	paired	0,92957 n.s. t=-0.09, DF=6							
Processes subset pipette withdrawn (Fig. 3c)	17,5	11,7	17,7	7		7										
Somata bolus loaded subset (Fig. 3e)	12,4	5,1	11,3	50		6	WSR	paired	5,34E-06 *** W=192, Z=-4.30							
Processes bolus loaded subset (Fig. 3e)	16,9	5,8	15,7	50		6										
Processes all individual (Fig. 3h)	16,9	7,2	16,3	29	131	25	MWU	unpaired								
Processes grouped by distance (5-15 m, Fig. 3j)	14,9	7,2	15,8	20	51	20	PS-TT	paired	0,00451 ** t= -3.20, DF=20							
Processes grouped by distance (15-25 m, Fig. 3j)	20,8	8,7	19,6	14	21	14										
Em (mV)	-84,8	0,4	-85,0	24		24										
**Determinants of Na+**	**Mean**	**SD**	**Median**	**n**	**n_p**	**N**										
Baseline Subset (Fig. 4a, b)	12,5	3,6	12,0	60		18	2S TT	unpaired	0,43597 n.s. t=0.78, DF=111							
Baseline TTX (Fig. 4a, b)	11,9	4,1	12,1	53		22										
Baseline Processes subset (Fig. 4a)	19,2	8,9	16,4	13	44	4	MWU	unpaired	0.174 n.s. U=901, Z=-1.36							
Baseline Processes TTX (Fig. 4a)	16,1	6,4	16,2	13	49	4										
Subset Baseline (Fig. 4c, same set as above)	13,0	7,4	11,2	186		7	WSR	paired	0,03 * W=108, Z=-2.16							
Subset + CBX (Fig. 4c)	20,0	13,3	18,4	134		7										
K+e: ISM Control (Fig. 4d)	2,5	0,1	2,4			8	WSR	paired	0,01563 ** W=0, Z=-2.28							
K+e: ISM CBX (Fig. 4d)	2,5	0,1	2,5			8										
Distance <50 µm (Fig. 4i)	-0,8	4,1	-1,1	92		4	VT	unpaired	<0.0001 *** F=0.31, DF=91							
Distance 50-100 µm (Fig. 4i)	-1,0	7,4	-1,2	272		4				0,01823 * F=0.75, DF=271						
Distance >100 µm (Fig. 4i)	-0,9	8,5	-1,6	280		4	VT	unpaired								
Baseline (0K only) (Fig. 4l)	16,2	8,2	14,8	24		4	WSR	paired	1,19E-07 *** W=0, Z=-4.27							
Peak (0K, 100 Na cap) (Fig. 4l)	69,1	31,9	73,8	24		4										
Baseline (0K only) + TTX (Fig. 4l)	11,8	4,7	12,6	14		5	PS TT	paired	8,87E-05 *** t= -5.58, DF=13							
Peak (0K, 100 Na cap) + TTX (Fig. 4l)	42,5	24,7	37,8	14		5										
Delta (0K) (Fig. 4m)	52,9	27,8	56,6	14		5	MWU	unpaired	0,01365 * U=86, Z=-2,47							
Delta (0K + TTX) (Fig. 4m)	30,7	20,5	26,1	14		5										
Resting Em (Fig. 4k)	-68,2	7,2	-70,1	9		7	2S TT	unpaired		0,20214 n.s. t=1.34, DF=13						
Hyperpolarized Em (Fig. 4k)	-76,4	5,7	-75,5	9		7	2S TT	unpaired			0,70911 n.s. t=0.38, DF=13					
Resting Em TTX (not illustrated)	-72,6	4,4	-72,9	6		5										
Hyperpolarized Em TTX (not illustrated)	-77,4	3,5	-77,4	6		5										
**TBOA**	**Mean**	**SD**	**Median**	**n**	**n_p**	**N**										
Neurons Delta TBOA (Fig. 5f)	67,0	23,0	69,3	96		4	MWU	unpaired	<1E-10 *** U=307, Z=-11.92							
Neurons Delta TBOA, CNQX and APV (Fig. 5f)	14,4	7,5	14,0	131		4										
Astrocytes Delta TBOA (Fig. 5f)	-4,6	4,0	-4,8	60		18	MWU	unpaired	6,83E-04 *** U=295, Z=-3.39							
Astrocytes Delta TBOA, CNQX and APV (Fig. 5f)	-1,7	3,4	-0,5	20		7										
K+e: ISM Baseline (Suppl. Fig 6d)	2,5	0,1	2,5			10	PS TT	paired	1,83E-04 *** t=-6.08, DF=9							
K+e: ISM 20 min TBOA (Suppl. Fig 6d)	4,8	1,2	4,8			10								1,70E-04 *** 2S TT unpaired t=-4.87, DF=16		
K+e: ISM 0K (Suppl. Fig 6d)	1,5	0,4	1,6			8									2,74E-06 *** PS TT paired t=13.60, DF=7	
rel. Area Baseline (Suppl. Fig 7b)	1,0	0,1	1,0	117		6	MWU	unpaired	2,54E-06 *** U=5311, Z=-4.7	0,59609 n.s. MWU unpaired U=3769, Z=-0.53	0,70553 n.s. MWU unpaired U=3966, Z=0.38					
rel. Area TBOA (Suppl. Fig 7b)	1,1	0,1	1,1	138		6							2,41E-06 *** MWU unpaired U=9459, Z=4.72			
rel. Area Baseline Subset (Suppl. Fig 7b)	1,0	0,1	1,0	76												
rel. Area APV, CNQX (Suppl. Fig 7b)	1,0	0,1	1,0	104		4	MWU	unpaired				0,33075 n.s. U=5666, Z=0.97				
rel. Area TBOA, APV, CNQX (Suppl. Fig 7b)	1,0	0,1	1,0	101		4										
**High K+**	**Mean**	**SD**	**Median**	**n**	**n_p**	**N**										
Somata Baseline subset for 10K (Fig. 6d)	12,3	3,6	11,6	29		8	PS TT	paired	2,01E-13 *** t=-13,05, DF=28							
Somata Decrease (Fig. 6d)	6,3	1,6	6,1	29		8										
Somata Delta (Fig. 6f)	-6,0	2,5	-6,4	29		8	MWU	unpaired		0,21074 n.s. U=235, Z=1.25	<0.0001 *** 2S TT unpaired t=-5.62, DF=20					
Somata Baseline TTX (Fig. 6e)	13,7	3,7	14,0	13		4	PS TT	paired	1,16E-07 *** t=11.08, DF=12							
Somata Decrease TTX (Fig. 6e)	6,6	2,1	7,2	13		4										
Somata Delta TTX (Fig. 6f)	-7,1	2,3	-6,3	13		4										
Processes Baseline subset for 10K (Fig. 6k)	23,3	6,5	24,8	5	17	5	WSR	paired	3,21E-04 *** W=153, Z=3.60							
Processes Decrease (Fig. 6k)	9,5	3,7	8,4	5	17	5										
Processes Delta (Fig. 6m)	-13,8	5,4	-13,8	5	17	5	MWU	unpaired		0,00861 ** U=120, Z=-2.63						
Processes Baseline TTX (Fig. 6l)	16,6	6,5	16,2	5	27	5	PS TT	paired	1,28E-12 *** t=12.66, DF=26							
Processes Decrease TTX (Fig. 6l)	7,0	3,4	7,2	5	27	5										
Processes Delta TTX (Fig. 6m)	-9,6	3,9	-9,2	5	27	5										
K+e: ISM Physiol (Suppl. Fig 6d)	2,6	0,2				8	PS TT	paired	4,47E-06 *** t= -12.64, DF=7							
K+e: ISM 10K (Suppl. Fig 6d)	7,3	0,9				8	2S TT	unpaired		0,77387 n.s. t= -0.29, DF=11						
K+e: ISM 10K + TTX (Suppl. Fig 6d)	7,1	0,5				5										
**Influence of different drugs on FL of SR101**	**Mean**	**SD**	**Median**	**n**	**n_p**	**N**										
SR101 BL (subset) (Suppl. Fig 5c)	4,1	0,1	4,1	27		5	2S TT	unpaired	0,45766 n.s. t= -0.75, DF=50							
SR101 after >15 min TTX (Suppl. Fig 5c)	4,1	0,1	4,1	25		5										
SR101 BL (subset) (Suppl. Fig 5d)	4,0	0,1	4,0	29		4	2S TT	unpaired	0,53627 n.s. t= -0.62, DF=57							
SR101 after >15 min CBX (Suppl. Fig 5d)	4,0	0,1	4,0	30		4										
SR101 BL (subset) (Suppl. Fig 5e)	3,9	0,3	3,9	24		5	PS TT	paired	0,22141 n.s. t= -1.26, DF=23							
SR101 after >15 min TFB-TBOA (Suppl. Fig 5e)	3,9	0,3	3,9	24		5										

MWU = Mann-Whitney Test, WSR= Wilcoxon rank sum test, PS TT = Paired Sample T-Test, 2S TT = Two Sample T-Test (Welch corr. if variance was not equal), VT = Test for Variance

p-value: *: 0.01 ≤ p < 0.05, **: 0.001 ≤ p <0.01 and ***: p < 0.001

	Degrees of freedom		red Chi2		Delta Chi2	AIC	F-Test	Significance
	1G	2G	1G	2G				
**Physiol**	21	19	51,20	13,77	813,6	2G	<0.0001	***
**TTX**	46	43	0,67	0,25	19,8	2G	<0.0001	***
**CBX**	46	43	2,73	1,37	66,9	2G	<0.0001	***
**Physiol SBFI**	8	8	3,53863	2,2453	10,3	2G	n/a	***
**Calibration**	Unconstrained	Original Fit	Unconstraine	Original Fit				
**Astrocyte Proce**	1	3	0,13	0,56	1,6	n/a	n/a	n.s.
**Astrocyte Soma**	1	3	8,77E-04	0,39771	1,2	n/a	n/a	n.s.

1G= single gaussian, 2G= double gaussian, AIC= Aikaike Information criterion
